# A novel miniaturized potentiometric electrode based on carbon nanotubes and molecularly imprinted polymer for the determination of lidocaine

**DOI:** 10.1007/s00604-024-06802-6

**Published:** 2024-11-15

**Authors:** Saad S. M. Hassan, Mahmoud Abdelwahab Fathy

**Affiliations:** Department of Chemistry, Faculty of Science, Ain Shams University, Abbasia, Cairo 11566 Egypt

**Keywords:** Potentiometric lidocaine selective electrode, Local anesthetic drugs, Screen-printed electrode, Molecular imprinted polymers (MIPs), Carbon nanotube solid contact

## Abstract

A novel miniaturized, solid-contact potentiometric screen-printed electrode was developed for highly sensitive and selective determination of lidocaine anesthetic. The electrode integrated single-walled carbon nanotubes as a solid-contact material and a molecularly imprinted polymer as a recognition sensory material. The performance characteristics of the electrode were evaluated and optimized to display a Nernstian slope of 58.92 ± 0.98 mV/decade over a linear concentration range of 4.53 × 10^−7^ to 6.18 × 10^−3^ mol/l within < 6 s. The detection limit was 7.75 × 10^−8^ mol/l (18.16 ng/ml) of lidocaine. The use of the molecularly imprinted polymer significantly enhanced the selectivity of the electrode, and carbon nanotubes increased the sensitivity, accuracy, and potential stability. The electrode was successfully used for determining lidocaine in pharmaceutical preparations and human urine. The results favorably compared with data obtained by liquid chromatography–tandem mass spectrometry.

**Graphical abstract:**

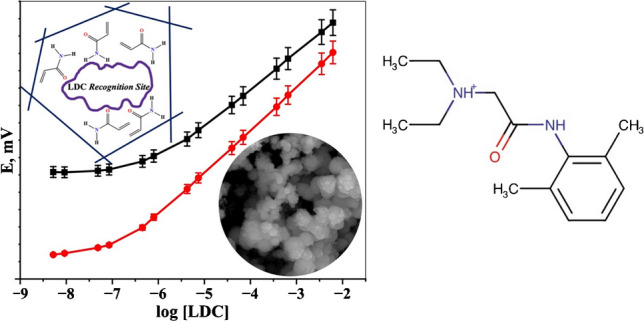

**Supplementary Information:**

The online version contains supplementary material available at 10.1007/s00604-024-06802-6.

## Introduction

Lidocaine (xylocaine or anestacon) is commonly used as a local topical anesthetic and anti-arrhythmic drug. It can also be used as an adjunct to tracheal intubation. It is widely employed in emergency departments, dental clinics, and primary care offices. Lidocaine belongs to the amide class of medications [1]. It functions by interrupting nerve signals, temporarily numbing the injected area [2]. Although its rapid onset of action makes it a favorable choice, this advantage can be overshadowed by potential side effects, including the risk of cardiac arrest and heightened nerve cell excitability, which can lead to chronic neurological symptoms such as seizures [3]. Consequently, monitoring the concentration of lidocaine is crucial to prevent overdose and the onset of adverse symptoms.

The selective monitoring of lidocaine is widely utilized in medical treatments and plays a significant role in detecting cases of poisoning and drug abuse. This is particularly important in clinical chemical analysis, forensic medicine, and emergency situations that necessitate the collection of evidence at crime scenes [4]. In this context, several analytical methods have been suggested for the determination of lidocaine, including spectrophotometry [5–7], electrochemiluminescence [8], high-performance liquid chromatography (HPLC) [9], liquid chromatography-mass spectrometry (LC/MS) [10], micellar liquid chromatography (MLC) [11], reversed phase liquid chromatography (RP-LC) [12], gas chromatography-mass spectrometry (GC/MS) [13], solid-phase microextraction gas chromatography (SPME-GC) [14], surface-enhanced Raman spectroscopy (SERS) [15], and voltammetry [16].

Although some of these techniques have high sensitivity, selectivity, and usually provide strong signals, they suffer from several limitations including the need for high-cost sophisticated equipment, specialized operational expertise, and time-consuming pre-treatment steps. These drawbacks make many of these methods less practical, particularly in situations requiring rapid analysis or dealing with a lot of sampling. Furthermore, some of these techniques are not directly applicable to colored and turbid sample solutions. To address these limitations, potentiometry has emerged as an optimal and simple solution. This method is cost-effective, simple in both preparation and application, and is characterized by its fast and stable response [17]. It also offers ease of miniaturization and does not require extensive technical expertise or pre-treatment.

These features, along with other compelling advantages, make potentiometric methods with ion selective electrodes, a highly desirable option. Potentiometric electrodes are further distinguished by their ability to be easily miniaturized for portable and point-of-care applications [18]. Unlike voltammetry, potentiometric electrodes allow direct analysis in complex biological matrices without the need for extensive sample preparation, making them highly practical for real-time monitoring in clinical and forensic settings. Moreover, their ability to function in complex matrices and ease of miniaturization are additional desirable benefits. Thus, several potentiometric electrode systems have been previously described for the determination of lidocaine [19–25]. However, these electrodes fall under the category of conventional ion-selective electrodes (ISEs), which require an inner filling solution and are relatively large size. Such designs come with several limitations, including evaporation of the inner-filling solution, a limited response of concentration range, and the difficulties in miniaturization, making them unsuitable for modern point-of-need or portable applications.

To address these limitations, solid-contact ion-selective electrodes (SC-ISEs) have been proposed as an effective solution. SC-ISEs are not only avoiding the need of an inner filling solution but also enhancing both the potential stability and portability of the electrode system. These systems use hydrophobic transducer materials, between the conductive substrate and the selective membrane, to improve ion-to-electron transduction [19]. Materials used for this purpose include conductive carbon-based materials [26], conductive polymers [27], and various nanomaterials [28–30]. In particular, single-walled carbon nanotubes (SWCNTs) are highly advantageous, as they exhibit high capacitance, which enhance electrode potential stability, and are chemically compatible with biomolecules, making them widely used in biotechnology and medicine.

In addition, single-walled carbon nanotubes (SWCNTs) offer several advantages over multi-walled carbon nanotubes (MWCNTs) and other carbon-based materials specially for providing a more efficient and direct pathway for electron transfer, reducing the trapping of redox-active species which interfere with the ion-to-electron transduction process. In contrast, the multi-layered structure of MWCNTs increases the possibility of encapsulating redox species [31], which hinder the ion-to-electron transduction process, affecting the short- and long-term potential stability. Moreover, the weaker interfacial adhesion between MWCNTs and the ion-selective membrane (ISM) leads to polymeric membrane delamination [32]. Consequently, SWCNTs are usually preferred for applications in potentiometric solid-contact ion-selective electrodes (SC-ISEs) to offer high potential stability, efficient ion-to-electron transduction and to ensure accurate and reliable measurements.

On the other hand, molecularly imprinted polymers (MIPs) have emerged as powerful tools for potentiometric electrodes due to their highly selective “lock-and-key” recognition mechanism [33]. This mechanism enables MIPs to function as precise sensing receptors for various compounds [34]. The MIP-based approach offers distinct advantages over traditional ion-pair complexes previously used for fabrication of lidocaine potentiometric electrodes, particularly in terms of selectivity, stability, and accuracy.

In this study, a novel miniaturized solid-contact potentiometric screen-printed electrode is developed for the determination of lidocaine (LDC). The electrode incorporates both SWCNTs as a solid-contact material and a LDC-MIP as a sensory material specific to lidocaine. The integration of these materials provides several key advantages, including enhanced sensitivity, fast response times, high potential stability, and exceptional selectivity for LDC. Moreover, the miniaturized design allows the electrode to be easily incorporated into portable, point-of-care diagnostic devices, and making it a highly practical solution for clinical and forensic applications.

## Materials and methods

### Materials and reagents

All chemicals and reagents used were of analytical grade with purity not less than 98%. Freshly deionized water (18.2 MΩcm specific resistance) using the Milli-Q PLUS reagent-grade water system (Millipore, Burlington, MA, USA) was used. Acrylamide monomer (AAm), benzoyl peroxide (BPO), ethylene dimethacrylate cross-linker (EDA), single-walled carbon nanotubes (SWCNTs, average diameter 2 nm), confirmed by transmission electron microscopy (Fig. S1), were used as solid-contact material, acetonitrile (MeCN), lidocaine hydrochloride (C_14_H_22_N_2_O. HCl), procaine hydrochloride (C_13_H_20_N_2_O_2_.HCl), tetracaine (C_15_H_24_N_2_O_2_), bupivacaine hydrochloride (C_18_H_28_N_2_O·HCl), mepivacaine hydrochloride (C_15_H_22_N_2_O.HCl), dibucaine hydrochloride (C_20_H_29_N_3_O_2_.HCl), and monoethylglycinexylidide (C_12_H_18_N_2_O) were purchased from Sigma-Aldrich (St. Louis, MO, USA). High molecular weight poly(vinyl chloride) (PVC), dioctyl phthalate plasticizer (DOP), and tetrahydrofuran (THF) were obtained from Fluka (Ronkonkoma, NY, USA). The screen-printed gold electrode (model AUSE100) was purchased from Zensor® (Taichung City, Taiwan). Working phosphate buffer solution (0.01 mol/l, pH 6.0) and a stock solution of LDC (0.1 mol/l) were freshly prepared and stored at 2 °C for further dilutions.

### Equipment

Fourier-transform infrared spectrometer (FTIR) with attenuated total reflection (ATR) (Alpha II, Bruker ABCO) was used. A field-emission scanning electron microscope (FESEM; QUANTA FEG 250, Netherlands) was used for the examination of the surface morphology of the electrode sensing membrane. Transmission electron microscopy (TEM) was carried out using JEOL-JEM-2100 electron microscope (Osaka, Japan). Constant-current chronopotentiometric (CP) measurements were conducted using a Metrohm potentiostat/galvanostat (Autolab, model 204 with NOVA 1.1 software, Metrohm Auto lab B.V., Utrecht, The Netherlands). All potentiometric measurements were carried out in 0.01 mol/l phosphate buffer at 25 ± 1 °C, using the developed screen-printed LDC MIP-based electrode and an Orion mV/pH meter Model 720/SA (Cambridge, MA, USA).

### Synthesis of imprinted lidocaine polymers

**Scheme 1:**
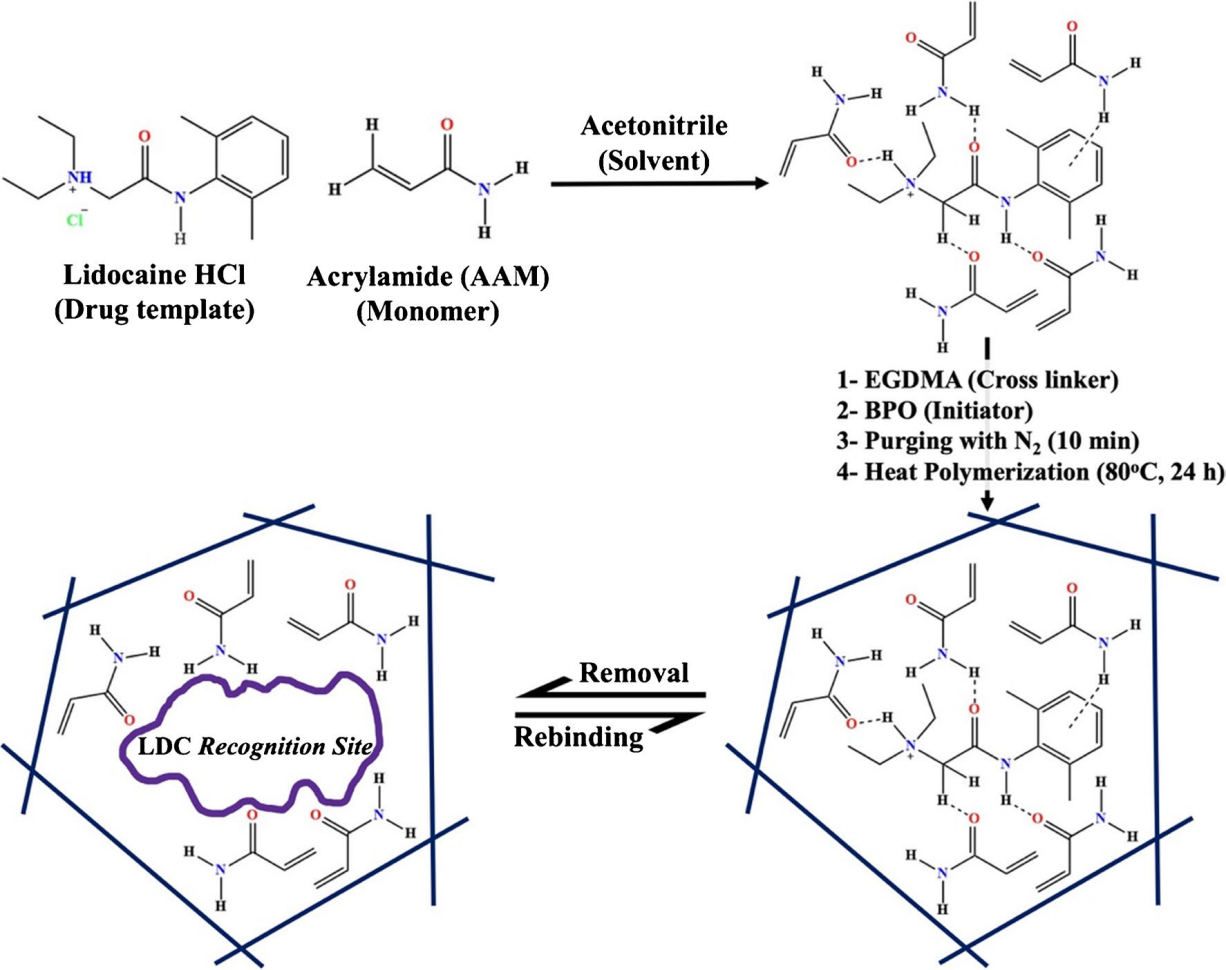
Schematic illustration of the steps involved in the molecularly imprinted polymerization process of the LDC drug based on acrylamide (AAm) as a functional matrix monomer

Lidocaine molecularly imprinted polymer (MIPs) was synthesized using the precipitation polymerization method [26] (Scheme 1). A mixture of 1.0 mol/l lidocaine hydrochloride as the imprinted template was mixed with, 3.0 mmol/l acrylamide (AAm), as the functional matrix monomer in a dark glass tube containing 5.0 ml of acetonitrile. The mixture was sonicated for 15 min to facilitate pre-complex formation between the drug and monomer molecules. Next, 5.0 mmol/l of ethylene dimethacrylate (cross-linker) was added to the mixture and allowed to react for 30 min. To initiate polymerization, 20 ml of acetonitrile and 50 mg of benzoyl peroxide (BPO) were added, respectively. Acrylamide monomer was previously used, satisfactorily, with some other drugs with similar structure [26, 34]. The monomer strongly interacts and binds with lidocaine, probably because both contain amine and carbonyl functional groups.

Nitrogen gas was allowed to pass into the polymerization mixture for 15 min to maintain an inert condition. The polymerization was carried out in an oil bath maintained at 70 °C overnight. The resulting polymer beads were filtered under vacuum, gently ground to fine powder, and purified using Soxhlet extraction with a methanol-acetic acid mixture (9/1, v/v) for 20 h to remove the non-reactive reactants and the drug template. The washed MIP beads were dried in vacuum at 70 °C overnight. Non-imprinted polymer (NIP) beads were prepared similarly without using the drug template.

### Construction of screen-printed lidocaine electrode

A screen-printed electrode (SPE) consisting of a 12 × 50 mm polycarbonate substrate with a 5-mm-diameter circular gold working electrode (model AUSE100, Zensor®, Taichung City, Taiwan) was employed. The SPE was immersed in a 1.0 mmol/l octanethiol solution for 2 h, and the gold layer was subjected to two coating cycles with 30 µl of SWCNTs, prepared by dispersing 10 mg of SWCNTs in 1.0 ml of THF. Using a smaller amount of SWCNTs resulted in a decrease in the double-layer capacitance, negatively affecting the short-term potential stability of the electrode. In contrast, increasing the amount of SWCNTs led to a sub-Nernstian response due to competition between the ion-to-electron transduction and the adsorption of the sensory material on the solid-contact layer, which hindered the ion sensitivity of the selective membrane. A plasticized PVC cocktail was prepared by dissolving. 4 mg of either LDC-MIP or NIP beads, 46.4 mg of PVC, 1 mg of NaTPB, and 185.2 mg of DOP in 2.8 ml of THF. Two portions, each containing 30 µl of the membrane cocktail, were drop-cast onto the SWCNTs layer and allowed to dry overnight, as illustrated in Scheme S1. The electrode was conditioned in a 1.0 mmol/l lidocaine hydrochloride solution for 24 h to allow the organic sensing/plasticizer in the membrane to get in equilibrium with the aqueous and test solution allowing the potential reading to stabilize within ± 0.5 mV/h before use.

### Potentiometric measurements

The screen-printed working electrode (SPE), in conjunction with an Orion Ag/AgCl double-junction reference electrode filled with 10% (w/v) KNO_3_, was used for measurements. Calibration of the electrode involved transferring of 0.5–1.0 ml aliquots of freshly prepared aqueous lidocaine solution with concentrations ranging from 1.0 × 10^−1^ to 1.0 − 10^−7^ mol/l to a 25-ml beaker containing a 10 ml aliquot of 0.01 mol/l phosphate buffer solution of pH 6.0. Potential readings were recorded after stabilization to ± 0.5 mV. The electromotive force (EMF) was plotted against the logarithm of lidocaine concentrations. The selectivity coefficients (${K}_{LDC,j}^{pot}$) of some interfering species commonly associated with lidocaine in pharmaceutical formulations or present as major metabolites in human biological fluids (i.e., glycylxylidide and monoethylglycinexylidide) [35] were determined using the separate solution method (SSM) [36].

### Analytical applications

Local anesthetic injection ampoules containing 10–20 mg/ml lidocaine were analyzed by the proposed electrode. The contents of 10 ampoules were mixed; a 1.0 ml aliquot was diluted with 9.0 ml of phosphate buffer solution of pH 6; and the content of lidocaine was measured as described above. The performance and response of the proposed LDC-MIP electrode in synthetic urine samples containing 5.0 to 20.0 µg/ml lidocaine were examined. The synthetic urine background matrix was prepared to closely mimic the ionic composition of the human urine. The following components were mixed and dissolved in 1.0 l of nanopure water 0.651 g/l calcium chloride dihydrate (CaCl_2_·2H_2_O), 0.651 g/l magnesium chloride hexahydrate (MgCl_2_·6H_2_O), 4.6 g/l sodium chloride (NaCl), 2.3 g/l sodium sulfate (Na_2_SO_4_), 2.8 g/l potassium dihydrogen phosphate (KH_2_PO_4_), 1.6 g/l potassium chloride (KCl), 1.0 g/l ammonium chloride (NH_4_Cl), 25.0 g/l urea, and 1.1 g/l creatine [37]. Measurements with a reference liquid chromatography–tandem mass spectrometry (LC–MS–MS) method were conducted in parallel for comparison [38].

## Results and discussion

### Molecularly imprinted lidocaine polymer

Molecularly imprinted polymer (MIP) for the lidocaine drug and non-imprinted polymer (NIP) were synthesized and characterized. The polymers were examined by Fourier-transform infrared (FTIR) spectroscopy and by field emission scanning electron microscopy (FESEM). The binding affinity of lidocaine drug towards the MIP and NIP beads was also investigated.

#### FTIR

The Fourier-transform infrared spectroscopy (FTIR) spectrum of pure lidocaine (Fig. S2a) revealed a sharp peak at 1720.8 cm^−1^, attributed to the amide C = O group. Peaks appeared at 2978.9–2828.9 cm^−1^ were due to aliphatic C–H vibration modes. Two peaks at 3362.9 and 1480.1 cm^−1^ were observed, corresponding to N–H vibration modes [39]. The FTIR spectrum of LDC-imprinted polymer (MIP) beads (Fig. S2b) showed two characteristic peaks at 1728.8 and 1488.2 cm^−1^ with a noticeable band shift and reduced intensity compared to those obtained with the pure LDC drug. This indicated an interaction between the oxygen atom and the amide group of both the monomer (AAm) and LDC molecules.

The FTIR spectra of the LDC free molecularly imprinted polymer (MIP) and the non-imprinted polymer (NIP) are nearly identical with complete disappearance of the characteristic peaks of the LDC drug in the LDC free MIP, as shown in Fig. S2c, d), respectively. Both spectra exhibited a broad band around 3457.5 cm^−1^, assigned to N–H stretching and the presence of a characteristic peak due to the amide carbonyl group of the acrylamide monomer around 1674.4 cm^−1^. Additionally, two sharp peaks at 1729.4 and 1154.6 cm^−1^ corresponding to the stretching vibrations of -C = O and –C–O groups, originating from the use of ethylene dimethacrylate as a polymer cross-linker. These results confirmed the successful LDC imprinting process using acrylamide as a functional matrix monomer.

#### FESEM

Field emission scanning electron microscopy (FESEM) was employed to investigate the surface morphology of the prepared polymers and to provide insights into the distribution of particle sizes of both the molecularly imprinted and non-imprinted polymers. The FESEM micrograph of the LDC-free MIP beads and the particle size distribution histogram (Fig. S3a) revealed asymmetrical, irregular and shattered beads with an average particle size of 45.5 nm. However, the non-imprinted polymer (Fig. S3b) exhibited spherical and uniform beads with an average particle size of 68.1 nm. These morphological differences resulted from the lack of specific LDC binding sites in the NIP beads and confirming the imprinting process in the MIP beads. The smaller average particle size of the MIP beads, in comparison with the NIP beads, caused an increase in the polymer surface area and enhanced the number of binding recognition sites for LDC drug.

#### MIP binding affinity and Scatchard analysis

Scatchard analysis of LDC-free MIP and NIP beads was conducted. A fixed amount of 15 mg portion of either MIP or NIP bead was added to a 10 ml aliquot of different LDC concentrations, ranging from 0.01 to 1.5 mmol/l. The mixtures were stirred for 30 min, followed by a 24-h incubation period to achieve equilibrium. After centrifugation, the solid polymer was separated and the excess LDC in solution was measured using liquid chromatography–tandem mass spectrometry (LC–MS–MS) method [38]. The binding capacity *Q*_*e*_ was calculated using Eq. (1).

1 $${Q}_{e} = ({C}_{i}- {C}_{f}){V}_{s}/m$$

where *Q*_*e*_ (μmol/g) was the binding capacity at equilibrium; *C*_*i*_ and *C*_*f*_ (μmol) were the initial and free concentrations of LDC, respectively. *V*_*s*_ (ml) was the volume of the mixture, and *m* (g) was the polymer mass. The binding characteristics were further assessed by Scatchard analysis using Eq. (2).

2 $${Q}_{e}/{C}_{f} = ({Q}_{\text{max}} - {Q}_{e})/{K}_{D}$$

where *Q*_max_ (μmol/g) was the maximum apparent binding capacity, and *K*_*D*_ was the equilibrium dissociation constant at the binding site.

**Fig. 1:**
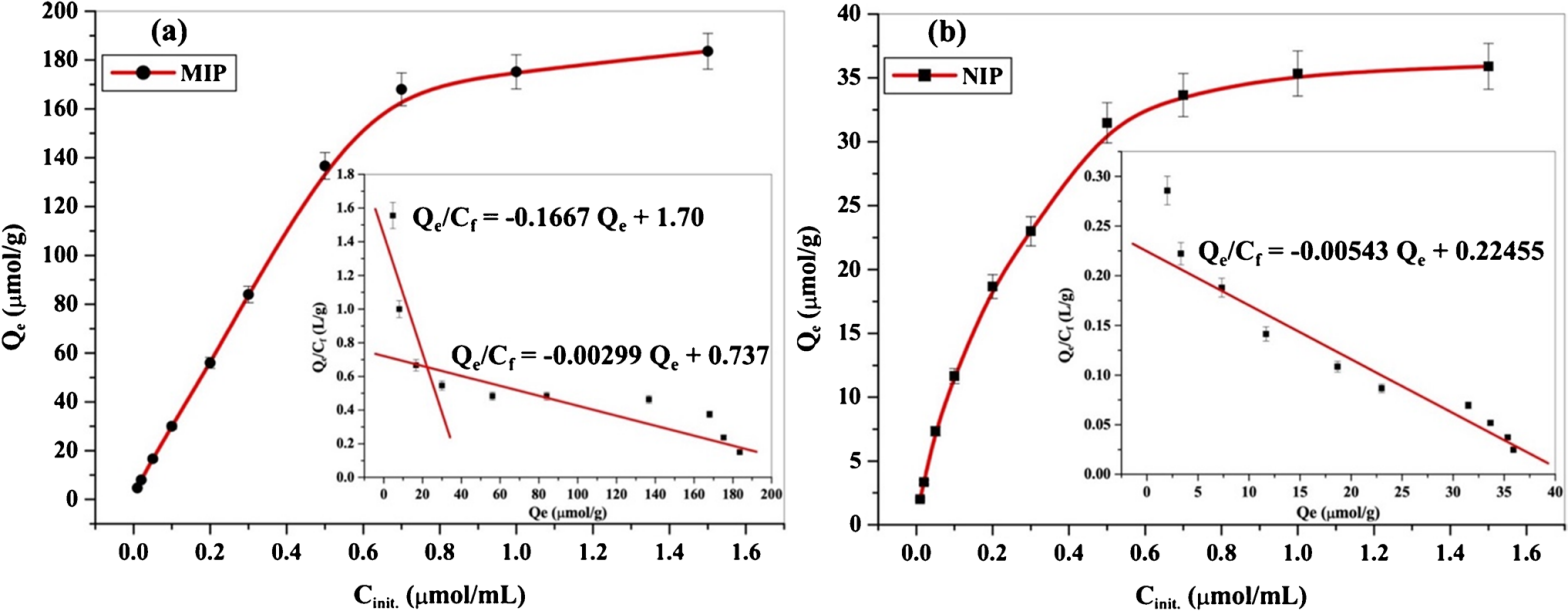
Binding isotherms with inserted Scatchard plots of (**a**) LDC-free MIP and (**b**) NIP. (Conditions: 15.0 mg of either MIP or NIP beads; *V* = 10.0 ml; *t* = 25 °C)

The binding isotherm plot for MIP beads (Fig. 1a) showed a notable increase in the binding capacity at the equilibrium (*Q*_*e*_) compared to NIP (Fig. 1b). This indicated a high affinity between MIP beads and the LDC drug molecules, confirming a successful molecular imprinting process and the formation of LDC recognition sites within the MIP beads. The Scatchard plot of MIPs (inserted in Fig. 1) revealed two linear regression lines with distinct slopes, indicating the presence of two different binding sites for LDC on the MIP beads [40]. The high-affinity line (left part) displayed *K*_*D*_ and *Q*_max_ values of 6.0 ± 0.31 μmol/l and 10.19 ± 0.52 μmol/g, respectively. The low-affinity line (right part) exhibited values of 334.45 ± 78.11 µmol/l and 246.54 ± 57.58 µmol/g, respectively. In contrast, NIP beads exhibited a single linear regression line with *K*_*D*_ and *Q*_max_ values of 184.16 ± 16.46 μmol/l and 41.35 ± 3.69 μmol/g, indicating a lower binding affinity of LDC to the polymer beads. It can be seen that MIP beads showed higher binding capacity (*Q*_*e*_) than NIP beads, regardless of the sample volume. In this study, we focused on a fixed volume to examine the molecular recognition performance of MIP beads and the higher affinity of lidocaine molecules towards MIP compared to NIP.

### Potentiometric performance characteristics

**Fig. 2:**
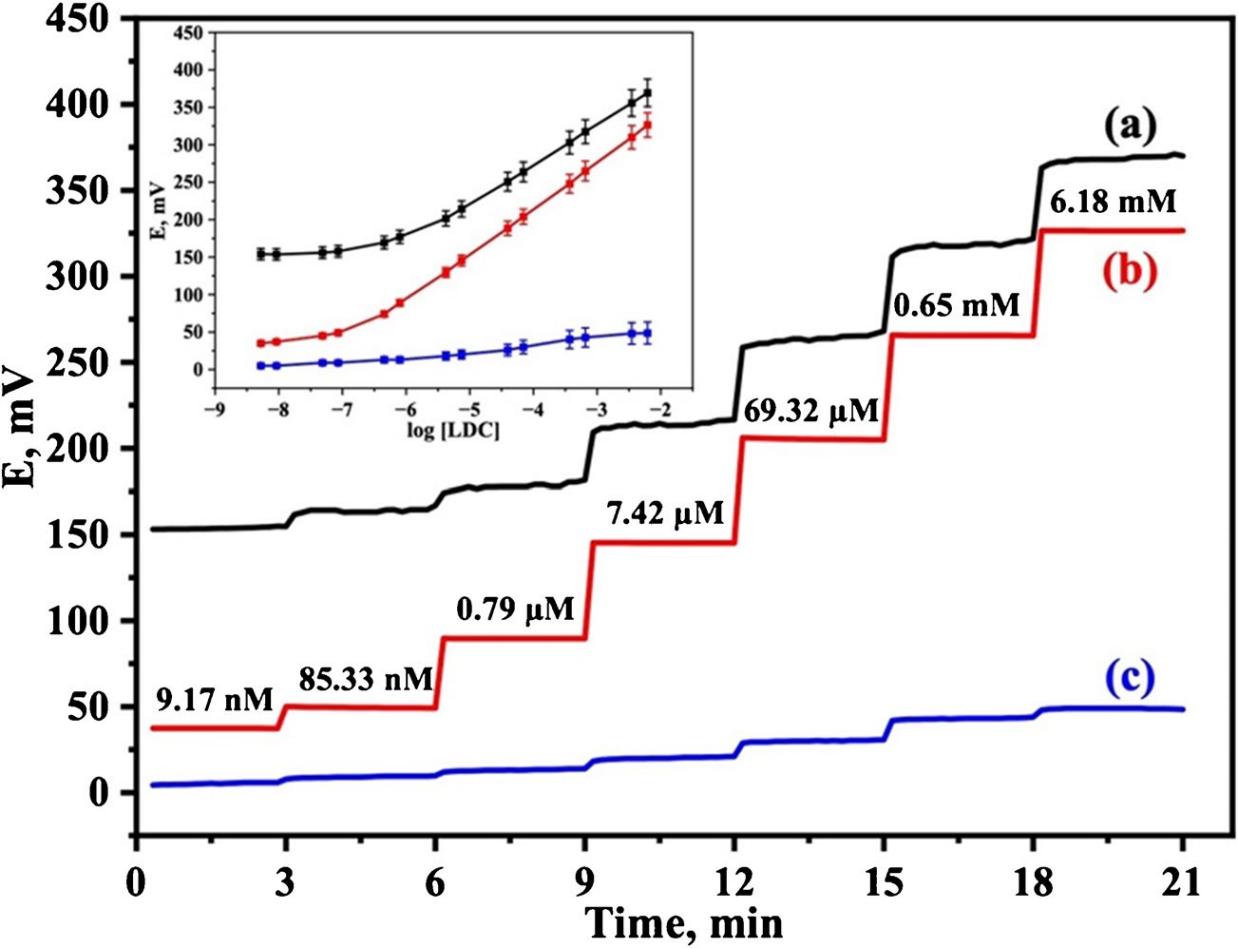
Response time of the proposed LDC-MIP electrode: (**a**) before and (**b**) after modification with SWCNTs, and (**c**) for the LDC-NIP electrode. (Inset: equivalent calibration curves)

The potentiometric response of the proposed screen-printed LDC electrode, based on a molecularly imprinted polymer (MIP), was evaluated and compared both before and after modification with the single-walled carbon nanotubes (SWCNTs) as a solid-contact transducing material using the IUPAC recommendations [41]. Calibration plots were constructed using a measuring cell containing a 10.0 ml aliquot of a 0.01 mol/l phosphate buffer solution of pH 6.0. Aliquots of lidocaine (LDC) solutions (0.5 to 1.0 ml) with concentrations of 1.0 × 10^−1^ to 1.0 × 10^−7^ mol/l were sequentially added, and the corresponding potentials were recorded (Fig. 2).

The LDC-MIP modified electrode exhibited a Nernstian slope of 58.92 ± 0.98 mV/decade (*n* = 3, *R*^2^ = 0.9998), a linear response range of 4.53 × 10^−7^ to 6.18 × 10^−3^ mol/l, a lower detection limit of 7.75 × 10^−8^ mol/l, and a response time of less than 6 s. The detection limit was calculated according to IUPAC guidelines [41], i.e., the concentration at the intersection of the extrapolated linear segments from the midrange and final low concentration level segments of the calibration plot. The rapid response time was attributed primarily to the use of single-walled carbon nanotubes (SWCNTs), which provide minimum trapping of redox-active species, accelerating the ion-to-electron transduction.

In contrast, the unmodified electrode displayed a sub-Nernstian slope of 49.42 ± 1.83 mV/decade (*n* = 3, *R*^2^ = 0.9942) over a narrower linear range from 4.22 × 10^−6^ to 6.18 × 10^−3^ mol/l, with a detection limit of 6.80 × 10^−7^ mol/l and a response time of 60 s, probably due to the absence of SWCNTs and the less efficient electron transfer. An electrode based on a non-imprinted polymer (NIP) showed a non-Nernstian calibration slope of 11.34 ± 2.45 mV/decade (*n* = 3, *R*^2^ = 0.9738) over a linear range of 3.45 × 10^−4^ to 6.18 × 10^−3^ mol/l. These results further confirmed the specific interaction between LDC drug and the molecularly imprinted polymer.

The MIP-PVC membrane compositions of the modified LDC screen-printed electrode were optimized through extensive testing of different amounts of the MIP sensory material in the PVC membrane. The PVC membrane cocktail was consisted of 0–6 mg of MIP beads, 46.4 mg of PVC, 1.0 mg of NaTPB, and 185.2 mg of DOP. Optimum and stable responses are obtained with a PVC membrane containing 4 mg MIP beads, revealing a Nernstian slope of 58.92 ± 0.98 mV/decade and a lower detection limit of 7.75 × 10^−8^ mol/l, as summarized in Table S1. However, using smaller amounts of MIP beads (ranging from 3 mg to blank, i.e., without MIP) resulted in a non-Nernstian slope with higher detection limits, thereby reducing the sensitivity of the electrode. Conversely, using larger amounts of MIP beads (5–6 mg) led to a sub-Nernstian response. Moreover, shifting from DOP (dielectric constant = 5.1) to *o*-NPOE (higher dielectric constant = 23.9) plasticizer [42, 43] resulted in a sub-Nernstian slope, likely because the higher ion mobility facilitated by *o*-NPOE caused an increase of the interactions between LDC-MIP and SWCNTs solid-contact layer. Such interaction led to adsorption and reduction of the electrode performance. Therefore, the optimum composition (4.0 mg MIP, 46.4 mg PVC, 1.0 mg NaTPB, and 185.2 mg DOP) was selected for further studies.

#### Influence of pH

The potential stability of the proposed modified electrode was tested using two concentrations of lidocaine (1.0 × 10^−3^ and 1.0 × 10^−4^ mol/l). The pH of the test solution was adjusted using small volumes of 0.1 mol/l HCl and/or NaOH. A consistent and stable potential reading was obtained over the pH range 4.5 to 7.0 (Fig. S4). Positive potential drift was observed at pH < 4.5, attributed to the interference of H^+^ ions and a negative potential drift occurred at pH > 7.0, attributed to deprotonation of the lidocaine tertiary amine, (LDC p*K*a = 7.9) [35]. All measurements were conducted in a 0.01 mol/l phosphate buffer solution at pH of 6.0.

#### Electrode selectivity

The standard separate solutions method (SSM) was used to examine the selectivity of the proposed LDC-MIP electrode towards some interfering cationic inorganic species, local anesthetics such as procaine, tetracaine, bupivacaine, mepivacaine, and dibucaine, as well as two major active metabolites of lidocaine [35] namely, glycylxylidide (GX) and mono-ethylglycinexylidide (MEGX). The selectivity coefficients ($${K}_{LDC,j}^{pot})$$ were calculated using Eq. (3) [36].

3 $$\text{log }{{K}^{pot}}_{i,j} = \frac{{E}_{j}-{E}_{i}}{S} + \left(1 - \frac{{z}_{i}}{{z}_{j}}\right)\text{ log}{a}_{i}$$

where* E*_*i*_ and *E*_*j*_ were the potential readings of the primary LDC^+^ ion and the interfering ion at the same activity, and *z*_*i*_ and *z*_*j*_ represent their respective charges. *S* is the calibration slope. Although the matched potential method (MPM) can also be used, SSM method was simple and appropriate in the present case because lidocaine and related tested organic amines in the measurement medium (phosphate buffer of pH 6.0) were not present in the neutral form but in their monovalent cationic form.

The obtained selectivity coefficient values, shown in Table 1, indicate significantly high selectivity of the proposed LDC-MIP-based electrode towards LDC over various inorganic ions and some commonly used amide local anesthetics. Furthermore, the proposed electrode demonstrated selectivity coefficient values in the range of 10^−2^ in the presence of the major lidocaine metabolites (i.e., glycylxylidide (GX) and monoethylglycinexylidide (MEGX). This high selectivity may be attributed to the “lock-and-key” mechanism offered by the molecular imprinting polymer (MIP), where specific recognition sites were formed within the polymer that match the size, shape, and functional groups of lidocaine. These tailored recognition sites allowed the electrode to specifically distinguish LDC from other local anesthetics and major metabolites.

**Table 1:** Selectivity coefficients (${K}_{LDC,j}^{pot}$) of LDC-MIP based electrode modified with SWCNTs

Interfering ion, *j*	log ${K}_{i,j}^{pot}$	${K}_{i,j}^{pot}$
Li^+^	− 3.96	1.11 × 10^−4^
Na^+^	− 3.36	4.41 × 10^−4^
K^+^	− 3.75	1.79 × 10^−4^
NH_4_^+^	− 2.88	1.33 × 10^−3^
Cs^+^	− 3.88	1.33 × 10^−4^
Cr^3+^	− 4.08	8.30 × 10^−5^
Ni^2+^	− 4.22	5.99 × 10^−5^
Co^2+^	− 3.89	1.28 × 10^−4^
Mg^2+^	− 4.11	7.76 × 10^−5^
Zn^2+^	− 4.99	1.02 × 10^−5^
Cu^2+^	− 3.96	1.10 × 10^−4^
Fe^2+^	− 4.06	8.79 × 10^−5^
Mn^2+^	− 4.20	6.26 × 10^−5^
Ca^2+^	− 4.39	4.11 × 10^−5^
Sr^2+^	− 4.66	2.21 × 10^−5^
Ba^2+^	− 5.02	9.64 × 10^−6^
Procaine*	− 3.18	6.67 × 10^−4^
Tetracaine*	− 3.06	8.79 × 10^−4^
Bupivacaine*	− 3.49	3.27 × 10^−4^
Mepivacaine*	− 3.38	4.21 × 10^−4^
Dibucaine*	− 3.10	7.94 × 10^−4^
Glycylxylidide (GX)*	− 1.94	1.14 × 10^−2^
Monoethylglycinexylidide (MEGX)*	− 1.69	2.06 × 10^−2^

*These compounds are in their monovalent cationic form at pH 6.0

#### Water layer test

**Fig. 3:**
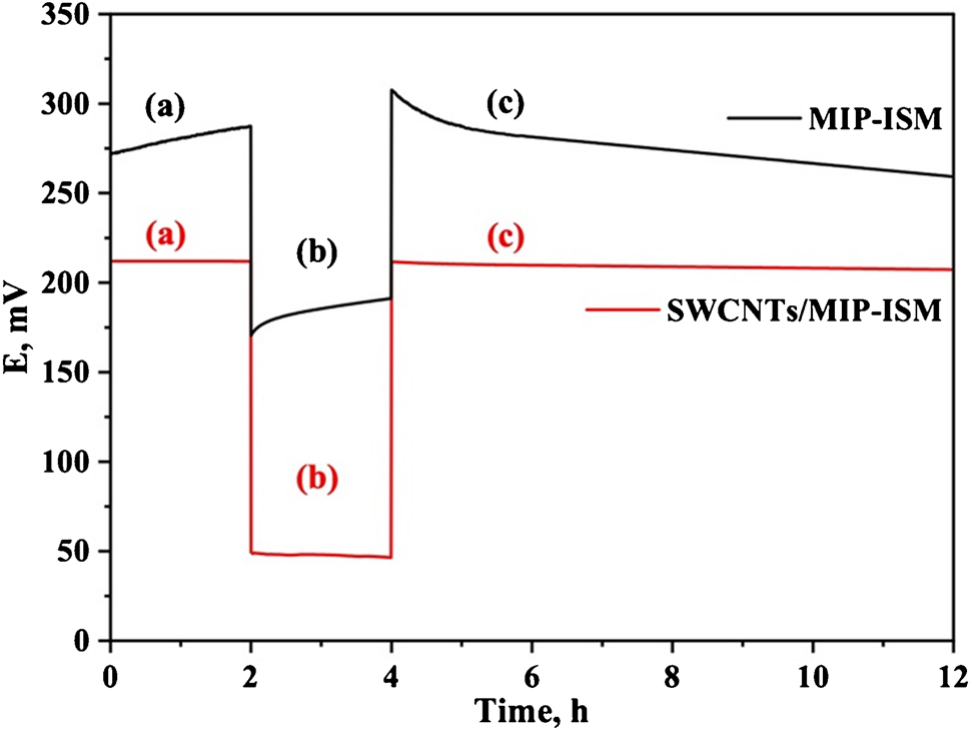
Water layer test of the LDC-MIP based electrode before and after incorporating SWCNTs as solid-contact material in (**a**) 0.1 mmol/l lidocaine, (**b**) 10 mmol/l phosphate buffer, and (**c**) reverting to 0.1 mmol/l lidocaine

One of the major problems of planar electrodes was the formation of a water layer between the conductive substrate base and the polymeric active film. This resulted in delamination of the PVC membrane from the electrode substrate surface and the formation of an O_2_/H_2_O half-cell, which significantly affect the long-term potential stability [44]. The use of SWCNTs as a solid-contact material was examined to reduce the water layer and to increase potential stability. The electrode was immersed before and after using SWCNTs in a 0.1 mmol/l lidocaine solution for 2 h. Subsequently, it is transferred to a 10 mmol/l phosphate buffer (pH 6.0) solution for another 2 h and then returned to a 0.1 mmol/l lidocaine solution again for an additional 8 h, and the electrode potential is measured during each cycle, as shown in Fig. 3.

The results in Table S2 reveal the effectiveness of SWCNTs in reducing the water layer due to their hydrophobic nature [45], resulting in an increase of the long-term potential stability. It can be seen that the use of SWCNTs resulted in a significant reduction of potential drift during the initial 2 h from 7.83 to − 0.03 mV/h, from 8.03 to − 0.96 mV/h during the next 2 h and finally from − 5.07 to − 0.49 mV/h during the last 8 h.

### Electrode potential stability

**Fig. 4:**
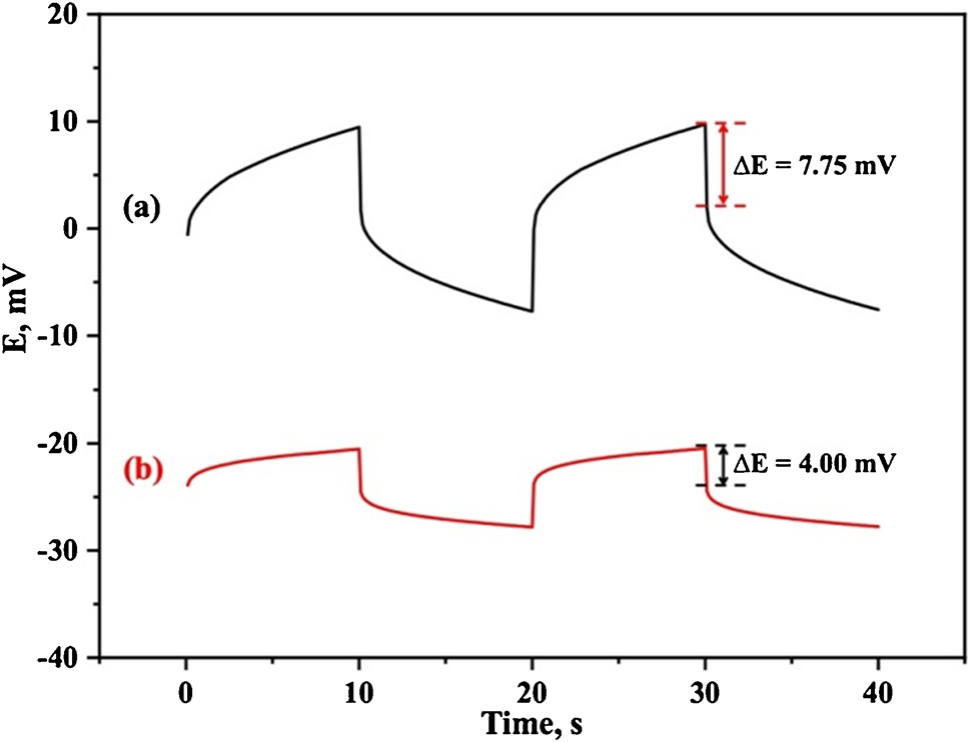
Chronopotentiogram of (**a**) MIP-ISM and (**b**) SWCNTs/MIP-ISM by applying a constant cathodic and anodic current of 20 nA for 10 s, repeated twice in 0.1 mol/l LDC solution

The potential stability of SC-ISEs was evaluated by examining the electrochemical characteristics of the ion-to-electron transducer layer. Chronopotentiometry was used to characterize the capacitance of the SWCNTs layer as a solid-contact transducer material after the deposition of the PVC selective membrane [46]. Chronopotentiometry measurements are performed by applying a constant cathodic and anodic current of 20 nA for a duration of 10 s, repeated twice in 0.1 mmol/l LDC solution, as shown in Fig. 4. Both the potential drift (*∆E/∆t*) and double layer capacitance (*C*_*d*_) were calculated using Eq. (4).

4 $$Potential \;drift = \frac{\Delta E}{\Delta t} = \frac{i}{{C}_{d}}$$

where *∆E/∆t* was the slope during the last 5 s of the chronopotentiogram; *i* the applied current (20 nA) and *C*_*d*_ the double layer capacitance.

The results obtained (Table S3), demonstrated a significant improvement in the short-term potential stability of the developed SC-SPE due to the presence of SWCNTs as a solid-contact material in LDC-MIP-based electrode, which reduced the bulk membrane resistance from 387.7 ± 3.1 to 199.8 ± 3.5 kΩ. In addition, a substantial increase in the double layer capacitance (*C*_*d*_) from 40.0 ± 0.08 to 138.3 ± 0.06 µF, with a specific capacitance of 461.2 ± 8.5 mF/g was observed. This significant reduction in membrane resistance, coupled with an increase in the double-layer capacitance, was attributed to the unique structure of SWCNTs, which increased the charge transport, ion-to-electron transduction, and contributed to the improved overall performance and stability of the electrode. These findings agreed fairly well with the previous literature data [46] confirming the proficiency of SWCNTs as a solid-contact ion-to-electron transducer suitable for use in the proposed LDC MIP-based electrode.

### Method validation

The method applied for determining lidocaine using the proposed LDC-MIP electrode, modified with SWCNTs, was validated using the directions of IUPAC [36]. ISO/IEC 17025, U.S. Pharmacopeia (USP), U.S. Food and Drug Administration (USFDA) standards [47] were also used for parameters connected to pharmaceuticals and not included in IUPAC directions. This comprehensive process encompassed precision, accuracy, trueness, within-day reproducibility, between-days variability, standard deviations, sensitivity, robustness, range, stability, durability, and limit of detection. These parameters were measured and calculated as described in our previous study [48]. The obtained results (Table 2) confirmed the analytical efficiency, reliability, accuracy, consistency, and applicability for quality control/quality assurance measurements.

**Table 2:** Method validation data obtained with LDC MIP electrode modified with SWCNTs

Parameter	Value
Slope, mV/decade	58.92 ± 0.98
Linear range, mol/l	4.53 × 10^−7^ to 6.18 × 10^−3^
Correlation coefficient, *R*^2^	0.9998
Detection limit, mol/l	7.75 × 10^−8^
Working pH range, pH	4.5 to 7.0
Double layer capacitance, µF	138.3 ± 0.06
Specific capacitance, mF/g	461.2 ± 8.5
Response time, s	< 6
Precision, (%)	0.6 ± 0.2
Accuracy (recovery), (%)	98.4 ± 0.5
Trueness, (%)	98.1 ± 0.6
Within-day reproducibility, (*CV*_w_) (%)	0.8 ± 0.2
Between-days variability (*CV*_b_), (%)	0.5 ± 0.1
Relative standard deviation, (%)	0.6 ± 0.1
Life span, (days)	60 ± 3

### Analytical applications

#### Determination of lidocaine in pharmaceutical products

The proposed modified LDC-MIP electrode was used for the direct potentiometric determination of lidocaine content in some local anesthetic injection ampoules containing 10 and 20 mg//ml. The contents of 5 lidocaine ampoules were diluted with 0.01 mol/l phosphate buffer solution of pH 6.0 and the potentiometric responses were measured and compared with a calibration graph. Lidocaine content of the same samples was assessed using a liquid chromatography–tandem mass spectrometry (LC–MS–MS) method [38]. The potentiometric results with the proposed modified electrode (average recovery 96.78%) were compared with the results obtained by the LC/MS–MS method (average recovery 97.63%). A close agreement within < 1% was obtained (Table 3). The *F*-test values were below 19.0, at a confidence level exceeding 95%. The proposed method was also used for monitoring lidocaine stability and ampoules homogeneity.

**Table 3:** Potentiometric determination of lidocaine in local anesthetic injection ampoules using the proposed LDC MIP electrode modified with SWCNTs

Pharmaceutical product	Labeled content	Found, mg/ml^a^				*F*-test^b^
		LC–MS–MS	Recovery, %	Proposed electrode	Recovery, %	
ASPEN® (CO, USA)	20 mg/ml	19.23 ± 0.18	96.2	18.91 ± 0.43	94.55	5.71
		19.32 ± 0.23	96.6	19.15 ± 0.29	95.77	1.59
HOSPIRA® (IL, USA)	10 mg/ml	9.99 ± 0.12	99.9	9.86 ± 0.16	98.60	1.78
		9.78 ± 0.34	97.8	9.74 ± 0.35	97.39	1.06

^*a*^Average of 3 measurements (*n* = 3)

^*b*^*F*-test value at a confidence level of 95% is 19.00

#### Analysis of lidocaine in synthetic urine samples

It has been reported that the level of lidocaine in human urine varied from 1 to 20 µg/ml depending on the treatment, such as laparoscopic surgery [49], intravenous infusion [50], and cholecystectomy [51]. For patients adhering to recommended therapy, lidocaine HCl levels ranged between 12 and 15 µg/ml [52]. Consequently, different concentrations of lidocaine at levels similar to those expected in patient urine (5–20 µg/ml), were spiked into synthetic urine samples. The mixtures were diluted (1:3) with a 0.01 mol/l phosphate buffer solution of pH 6.0) before potentiometric measurement using the proposed modified LDC-MIP electrode. The results obtained (Table 4) showed, an average recovery of 95.4 ± 1.9% of the spiked, indicating no matrix effect and no interference from the associated materials. Lidocaine in the test samples were also determined by LC–MS–MS for comparison. Close agreement between the chromatographic and the present potentiometric methods was obtained.

**Table 4:** Potentiometric determination of lidocaine (LDC) in synthetic urine samples using the proposed LDC-MIP based electrode modified with SWCNTs

Sample no	Spiked, µg/ml	Found, µg/ml^a^				*F*-test^a^
		LC–MS–MS	Recovery, %	Proposed electrode	Recovery, %	
1	5.0	4.86 ± 0.04	97.2 ± 0.8	4.68 ± 0.04	93.6 ± 0.8	1.56
2	10.0	10.08 ± 0.02	100.8 ± 0.2	9.53 ± 0.03	95.3 ± 0.3	2.25
3	20.0	20.43 ± 0.10	102.2 ± 0.5	19.46 ± 0.10	97.3 ± 0.5	1.69

^a^Average of 3 measurements (*n* = 3)

^b^*F*-test value at a confidence level of 95% is 19.00

### Advantages of the LDC electrode

A comparison of the main potentiometric performance characteristics of the proposed LDC electrode with some previously suggested potentiometric electrodes is shown in Table 5. Most of the previously suggested electrodes relied on the use of ion-pair complexes of lidocaine as ion exchangers. The proposed electrode displayed several advantages over many of these designs [20–23, 53, 54]. These included faster response (6 s), higher selectivity due to the use of MIP instead of the non-selective ion-pair complexes, along with a higher sensitivity down to 4.5 × 10^−7^ mol/l, and a lower detection limit of 7.7 × 10^−8^ mol/l. Finally, the use of a simple miniaturized planar solid-contact design eliminated the need for an inner filling solution and allowed the ease of integration into portable, point-of-care analytical devices for clinical and forensic applications.

**Table 5:** Main potentiometric characteristics of some previously reported lidocaine electrodes

Electrode type	Sensory material	Slope, mV/decade	Linear range, mol/l	Detection limit, mol/l	pH range	Response time for 10^−3^ mol/l, s	Ref
Liquid membrane	2-[Bis-octadecyl-sulfonic)-closo-decaborate	59.6 ± 0.7	7.0 × 10^−8^–1.0 × 10^−2^	2.0 × 10^−8^	3.6–7.4	20	[19]
Liquid membrane	Phosphotungstate	58.7 ± 0.2	2.6 × 10^−5^ – 1.0 × 10^−2^	1.9 × 10^−5^	2.0–6.0	10	[20]
Screen printed	β-Cyclodextrin tetraphenylborate	58.9 ± 0.7	6.2 × 10^−7^ − 1.0 × 10^−2^	1.0 × 10^−7^	2.0–8.0	6	[53]
Carbon paste		57.5 ± 0.9	1.0 × 10^−7^–1.0 × 10^−2^	6.2 × 10^−7^	2.0–7.5	4	
Liquid membrane	Dipicrylamine	58.2 ± 0.6	1.0 × 10^−4^–1.0 × 10^−1^	2.5 × 10^−5^	4.0–7.5	–-	[22]
	Dinonylnaphthalene sulfonate	57.3 ± 0.7	3.2 × 10^−5^–1.0 × 10^−1^	1.0 × 10^−5^	2.0–7.5		
Liquid membrane	Reineckate	29.0	3.0 × 10^−5^–1.0 × 10^−2^	1.0 × 10^–5^	3.0–6.5	60	[23]
Liquid membrane	Sulfathiazole	60.1 ± 0.2	1.0 × 10^−5^–1.0 × 10^−1^	6.0 × 10^−5^	5.0–9.5	< 10	[21]
Carbon cloth	Tetrakis(4-chlorophenyl) borate	57.1	1.0 × 10^−3^–2.0 × 10^−6^	2.0 × 10^−6^	2.0–8.0	< 10	[54]
Liquid membrane	Tetraphenylborate	56.0	3.1 × 10^−5^–1.2 × 10^−2^	–-	2.0–7.6	< 10	[24]
Coated wire	Tetraphenylborate	55.9 ± 0.9	1.0 × 10^−4^–1.0 × 10^−2^	–-	3.2–7.4	–-	[55]
Liquid membrane	Closo-hydridoborate	55.2 ± 0.2	1.0 × 10^−7^–1.0 × 10^−2^	3.5 × 10^−8^	4.0–7.5	–-	[25]
Screen-printed	Molecular imprinted polymer (MIP)	58.9 ± 0.9	4.5 × 10^−7^–6.1 × 10^−3^	7.7 × 10^−8^	4.5–7.0	< 6	This work

## Conclusion

A solid-contact potentiometric screen-printed electrode (SPE) for the selective determination of lidocaine (LDC) was successfully developed. The incorporation of single-walled carbon nanotubes (SWCNTs) as a solid-contact material and a molecularly imprinted polymer (MIP) as a sensory layer significantly enhanced the performance characteristics of the electrode. This design provided fast responses to lidocaine in pharmaceutical formulations and biological fluids. The suggested miniaturized, planar design eliminated the need for an inner filling solution, and enabled easy integration into portable, point-of-care devices for clinical and forensic applications. This novel electrode demonstrated higher sensitivity, potential stability, accuracy, and selectivity compared to many of those previously suggested.

## Supplementary Information

Below is the link to the electronic supplementary material.

Supplementary file1 (DOCX 979 KB)

## Data Availability

Data will be made available on request.
