# Supplementary material for: A novel miniaturized potentiometric electrode based on carbon nanotubes and molecularly imprinted polymer for the determination of lidocaine
Source: Mikrochim Acta. 2024 Nov 15;191(12):744. doi: 10.1007/s00604-024-06802-6 (PMC11564208; doi:10.1007/s00604-024-06802-6)
Supplement: Supplementary file 1 — Supplementary file1 (DOCX 979 KB) [file 604_2024_6802_MOESM1_ESM.docx]

**A novel miniaturized potentiometric electrode based on carbon nanotubes and molecularly imprinted polymer for the determination of lidocaine**

Saad S. M. Hassan^a,*^, Mahmoud Abdelwahab Fathy^a,*^

­­­­­

^a^*Department of Chemistry, Faculty of Science, Ain Shams University, Abbasia, Cairo 11566, Egypt*

* Corresponding author. *E-mail addresses:* [saadsmhassan@yahoo.com](mailto:saadsmhassan@yahoo.com) (S.S.M. Hassan),

[Mahmoud.abdelwahab@sci.asu.edu.eg](mailto:Mahmoud.abdelwahab@sci.asu.edu.eg) (M.A. Fathy).


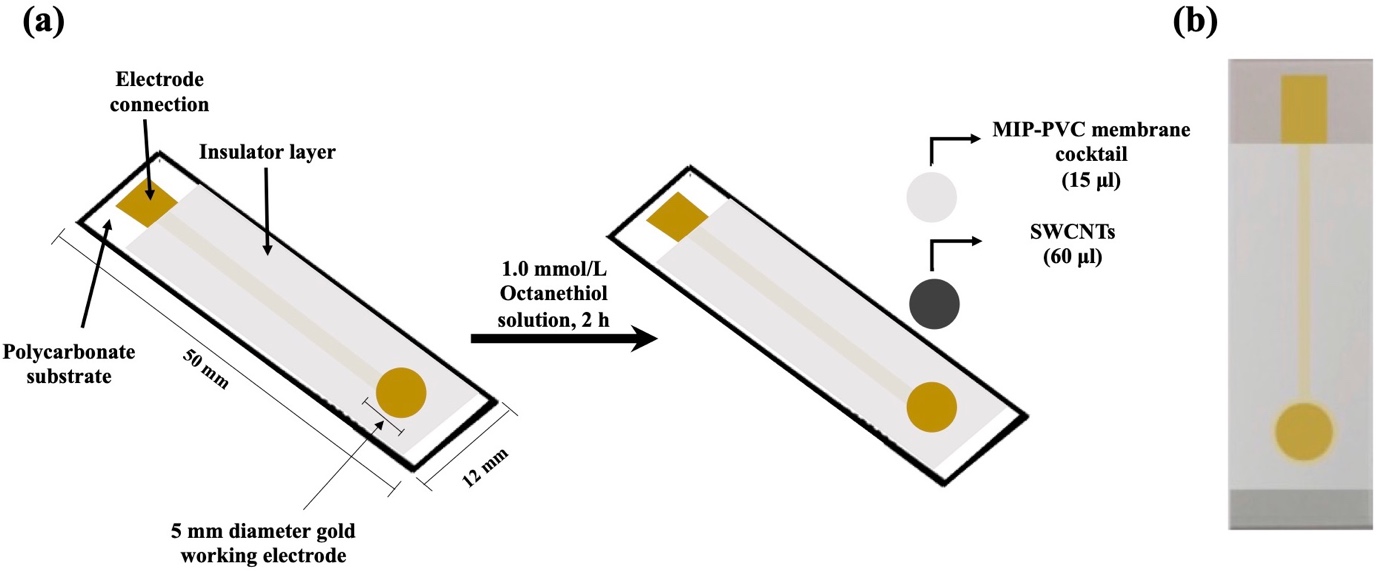


**Scheme S1.** Schematic representation of the construction process of the screen-printed electrode (SPE) for the determination of Lidocaine HCl, featuring (a) the fabrication procedure and (b) a photograph of the actual screen-printed electrode.


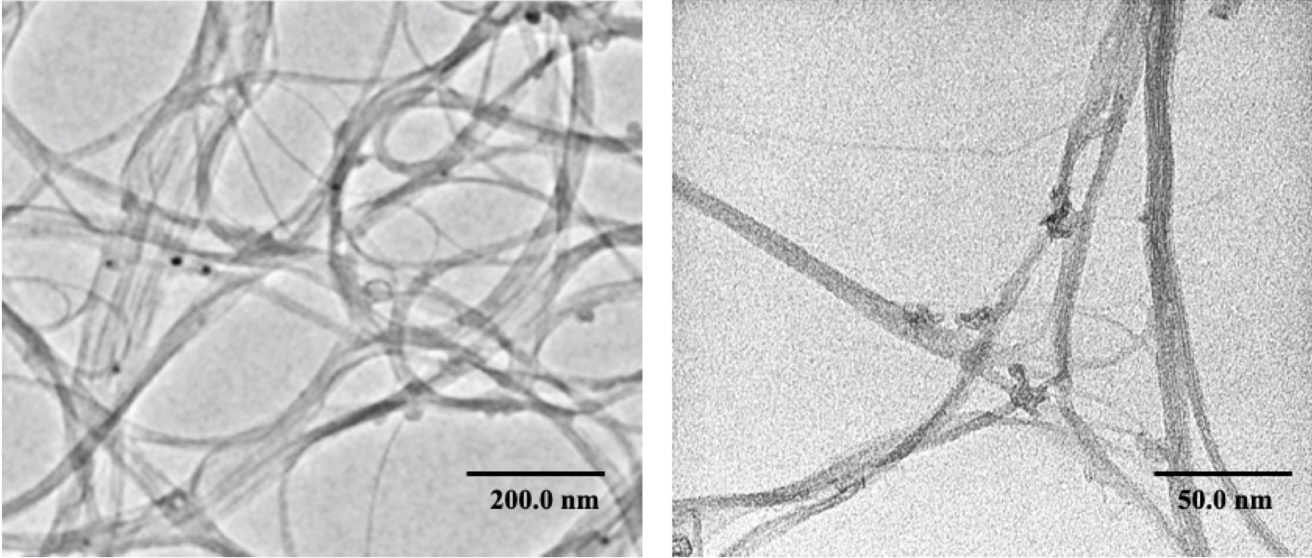


**Figure S1.** Transmission electron microscopy (TEM) images of single-walled carbon nanotubes (SWCNTs).


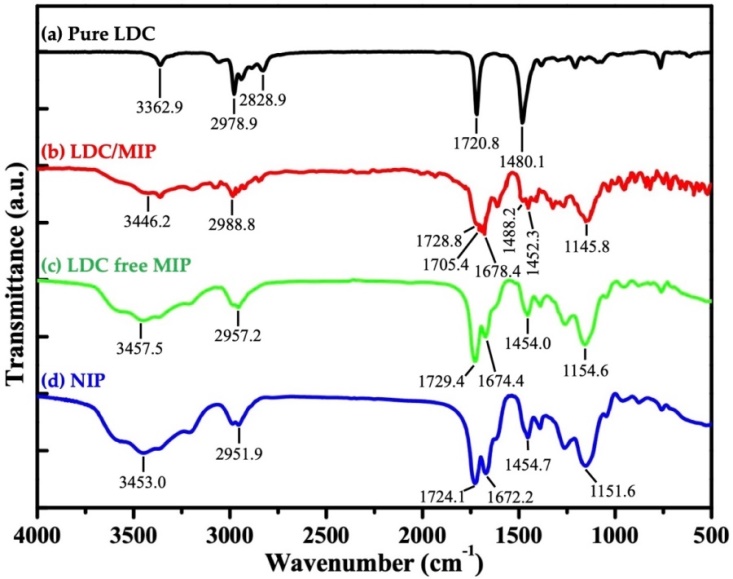


**Figure S2.** FTIR spectra of: (a) Pure LDC, (b) LDC/MIP (unwashed), (c) LDC free MIP (washed) and (d) NIP.


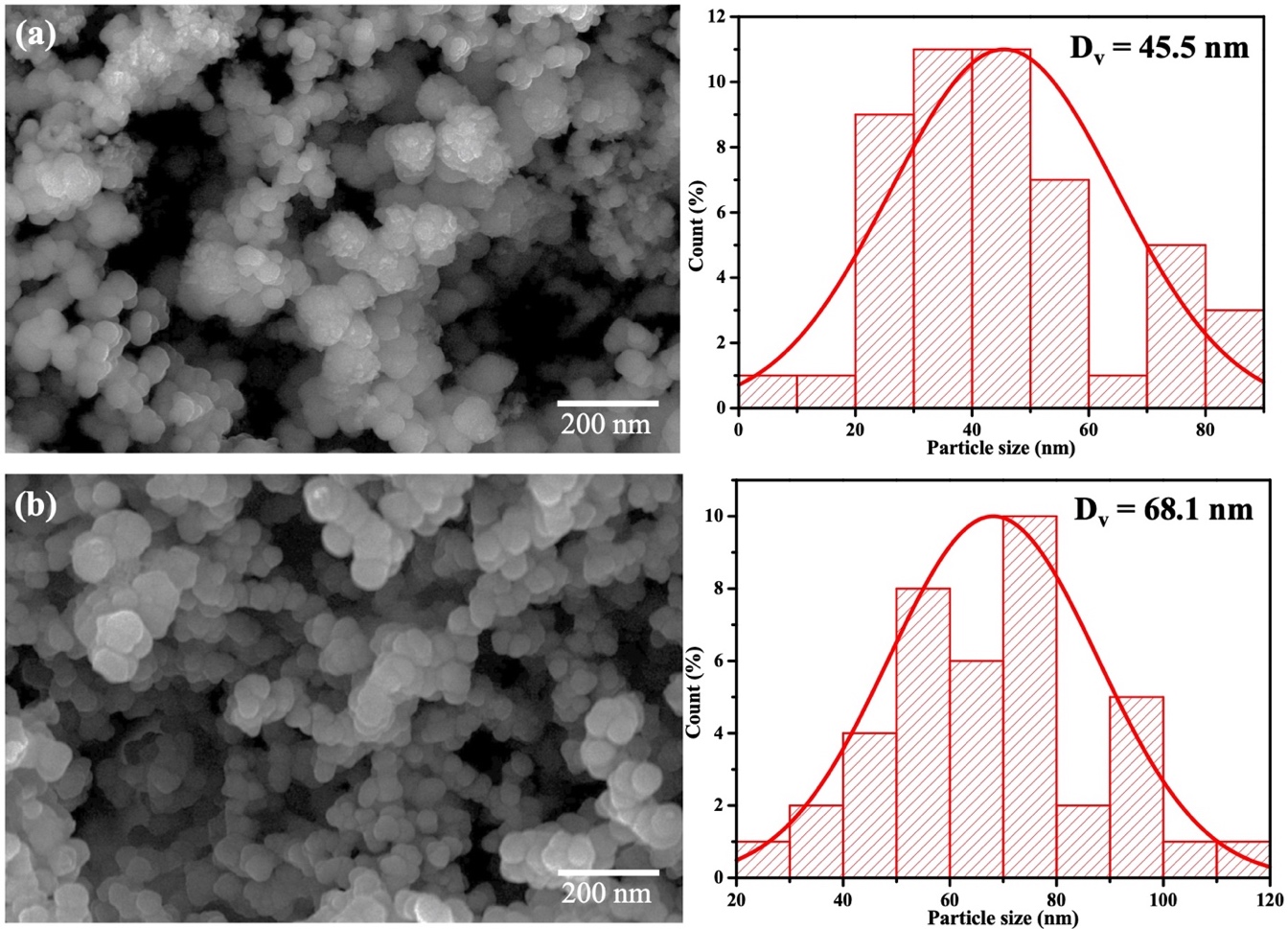


**Figure S3.** FESEM images and particle size distribution plots of (a) LDC free molecularly imprinted polymer (MIP) and (b) non-Imprinted polymer (NIP) beads.


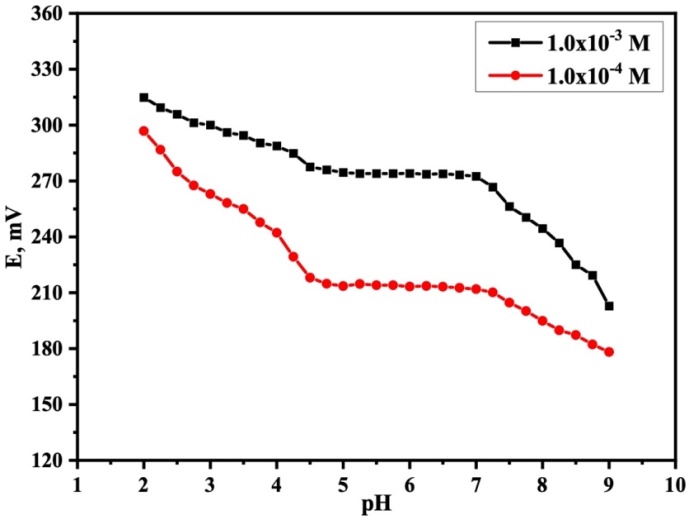


**Figure S4.** Influence of pH on potentiometric response.

**Table S1.** Optimization of the LDC-MIP membrane composition and its corresponding potentiometric characteristics.

| Sensor No. | PVC membrane composition (mg) | | | | | Potentiometric characteristics | | |
| --- | --- | --- | --- | --- | --- | --- | --- | --- |
|  | MIP | NaTPB | DOP | o-NPOE | PVC | Slope (mV/decade) | Detection limit  (mol/l) | Correlation coefficient  (R^2^) |
| 1 | --- | 1.0 | 185.2 | --- | 46.4 | 10.23 ± 2.34 | --- | 0.9713 |
| 2 | 1 | 1.0 | 185.2 | --- | 46.4 | 25.32 ± 2.65 | 1.35x10^-5^ | 0.9722 |
| 3 | 2 | 1.0 | 185.2 | --- | 46.4 | 42.35 ± 1.24 | 1.54x10^-5^ | 0.9723 |
| 4 | 3 | 1.0 | 185.2 | --- | 46.4 | 47.21 ± 0.32 | 1.54x10^-6^ | 0.9823 |
| 5 | 4 | 1.0 | 185.2 | --- | 46.4 | 58.92 ± 0.98 | 7.75x10^-8^ | 0.9998 |
| 6 | 5 | 1.0 | 185.2 | --- | 46.4 | 52.61 ± 1.42 | 6.57x10^-7^ | 0.9983 |
| 7 | 6 | 1.0 | 185.2 | --- | 46.4 | 48.78 ± 2.21 | 9.21x10^-7^ | 0.9836 |
| 8 | 4 | 1.0 | --- | 185.2 | 46.4 | 45.37 ± 0.91 | 1.72x10^-6^ | 0.9872 |

**Table S2.** Potential drift calculated through water layer test for the LDC-MIP sensor, both prior to and following the incorporation with SWCNTs as solid-contact material.

| **Solution** | **Duration, h** | **Potential drift, mV/h** | |
| --- | --- | --- | --- |
|  |  | MIP-ISM | SWCNTs/MIP-ISM |
| **Lidocaine**  **(0.1 mmol/l)** | 0 – 2 h | 7.83 ± 1.65 | -0.03 ± 0.002 |
| **Phosphate buffer**  **(10 mmol/l)** | 2 – 4 h | 8.03 ± 1.39 | -0.96 ± 0.05 |
| **Lidocaine**  **(0.1 mmol/l)** | 4 – 12 h | -5.07 ± 0.97 | -0.49 ± 0.02 |

**Table S3.** Data calculated from chronopotentiograms obtained in the presence and absence of SWCNTs layer, where a constant anodic and cathodic current of 20 nA was applied for 10 s in 0.1 mmol/l LDC solution.

| Parameter | MIP-ISM  (unmodified) | SWCNTs/MIP-ISM  (modified) |
| --- | --- | --- |
| ∆E, mV | 7.75 ± 0.002 | 4.0 ± 0.015 |
| Resistance, kΩ | 387.7 ± 3.1 | 199.8 ± 3.5 |
| Double layer capacitance, µF | 40.0 ± 0.08 | 138.3 ± 0.06 |
| Specific capacitance, mF/g | --- | 461.2 ± 8.5 |
